# Epidemiological and Clinical Parameters Features of Patients with Clonorchiasis in the Geum River Basin, Republic of Korea

**DOI:** 10.1155/2017/7415301

**Published:** 2017-04-26

**Authors:** Hee-Eun Shin, Myoung-Ro Lee, Jung-Won Ju, Byong-Suk Jeong, Mi-Yeoun Park, Keoung-Sook Lee, Shin-Hyeong Cho

**Affiliations:** ^1^Division of Malaria and Parasitic Diseases, Center for Immunology and Pathology, National Research Institute of Health, Centers for Disease Control and Prevention, Osong 28159, Republic of Korea; ^2^Department of Public Health, Okcheon-Gun Health Center, Okcheon 29032, Republic of Korea

## Abstract

This study was conducted to evaluate the infection rates of* Clonorchis sinensis* and laboratory findings in infected people. The 3,167 fecal samples, from nine villages in Okcheon-gun, were examined.

## 1. Introduction

*Clonorchis sinensis* is endemic in East Asian countries including China, Japan, Taiwan, Vietnam, and Korea [1, 2]. Worldwide, it is currently estimated that 15–20 million people are infected and 1.5–2.0 million people show symptoms or complications [3–5]. Although a rapid decrease in the overall prevalence of intestinal parasites has been observed in Korea, the prevalence of* C. sinensis* has remained high in riverside areas [6]. Nationwide surveys reported* C. sinensis* infection rates of 4.6% in 1971, 1.8% in 1976, 2.6% in 1981, 2.7% in 1986, 2.2% in 1992, 1.4% in 1997, 2.4% in 2004, and 1.9% in 2012 [7, 8]. Infection with the snail-transmitted trematode has been particularly frequent along several major Korean rivers, including the Geum, Nakdong, and Seomjin [9]. The rates of* C. sinensis* infection observed in the Geum River basin were 12.0% in 1981 [9], 4.6% in 2006 [10], and 3.1% in 2007 [11].

Freshwater fish is well-known as the major second intermediate host for* C. sinensis*, which can infect humans who ingest infected fish that is raw or undercooked [10]. The prevalence of infection is higher in males and increases with age in Korea [12]. Clonorchiasis patients suffer from symptoms such as epigastric pain, abdominal tenderness, fever, jaundice, and diarrhea [3]. Moreover, chronic infection with* C. sinensis* may cause more severe diseases such as cholangiocarcinoma [13]. The presence of the worms in the walls of the smaller bile ducts causes necrosis, inflammation, and biliary stricture through several mechanisms [14].

Early diagnosis and treatment are very important in preventing complications from clonorchiasis. The diagnosis depends mostly on detection of eggs in a patient's feces by either the formalin-ether concentration technique (FECT) or the Kato-Katz thick smear method [15]. Although molecular diagnostic techniques have been developed, early diagnosis of clonorchiasis remains difficult because subclinical infection with* C. sinensis* often occurs without obvious signs. Laboratory blood tests to look for changes in overall health are often part of a regular medical checkup. Individuals with abnormal blood test results should be further examined with a test suitable for early diagnosis. Previous studies reported that* C. sinensis* infected groups had differences in their serum alkaline phosphatase (ALP), aspartate aminotransferase (AST), alanine aminotransferase (ALT), *γ*-glutamyltranspeptidase (*γ*-GTP), and alpha-fetoprotein (AFP) test results and presented with peripheral eosinophilia [2, 16]. However, there is no diagnostic set of results from common blood and biochemical tests, except for those tests that can indicate compromised liver function.

In this study, we investigated sociodemographic and behavioral risk factors for* C. sinensis* infection in inhabitants living near the Geum River. We additionally evaluated the results of subjects' laboratory blood tests in order to look for possible early diagnostic indicators of clonorchiasis.

## 2. Materials and Methods

### 2.1. Study Site

**Figure 1:**
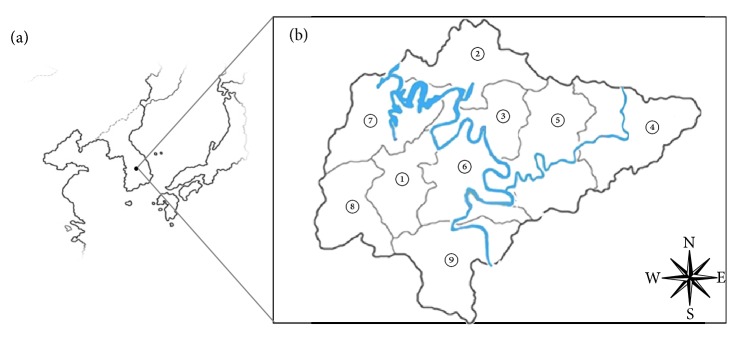
Geographic locations of the surveyed areas. Map shows (a) the Korean Peninsula and (b) the Okcheon-gun County of the Chungcheongbuk-do Province of Korea. The Geum River is shown in blue. Individual villages are shown on the map as follows: ① Okcheon-eup, ② Annae-myeon, ③ Annam-myeon, ④ Cheongsan-myeon, ⑤ Cheongseong-myeon, ⑥ Dongi-myeon, ⑦ Gunbuk-myeon, ⑧ Gunseo-myeon, and ⑨ Iwon-myeon.

The study was undertaken during the period of January to December 2013. A total of 3,167 inhabitants (1,227 males and 1,940 females) from nine villages in the Okcheon-gun County of the Chungcheongbuk-do Province of Korea were examined (Figure 1). The ages of the inhabitants ranged from 9 to 99 years, and the average age was 68.4 years (Table 1).

### 2.2. Fecal Examination

Stool specimens were collected in plastic stool containers and transferred to the laboratory of the Korea National Research Institute of Health during the period from February through May 2013. All participants were recruited for stool collection by a random household sampling method. Stool specimens were examined using the formalin-ether concentration technique (FECT) to assess the prevalence of parasitic infection, and the eggs per gram (EPG) were calculated using Stoll's egg counting method. Patients who tested positive for eggs (egg-positive) were considered infected, and those who tested negative for eggs (egg-negative) were considered uninfected.

### 2.3. Laboratory Examination

Blood samples (5 mL per individual) were collected from 97 participants using both 2-[2-[bis(carboxymethyl)amino]ethyl-(carboxymethyl)amino]acetic acid (EDTA) coated tubes and serum-separating tubes (SSTs). Samples were examined using 15 hematological parameters: hemoglobin (Hb), hematocrit (Hct), red blood cell count (RBC), white blood cell count (WBC), platelet distribution width (PDW), platelet counts, mean corpuscular volume (MCV), mean corpuscular hemoglobin (MCH), mean corpuscular hemoglobin concentration (MCHC), percent eosinophils (EOS), percent basophils, percent monocytes, percent neutrophils (NEUT), percent lymphocytes, and mean plasma volume (MPV). In addition, we analyzed 18 biochemical parameters using an automatic blood analyzer (Olympus AU 400): AST, ALT, lactate dehydrogenase (LDH), ALP, creatine phosphokinase (CPK), *γ*-GTP, total protein, albumin, total cholesterol, high-density lipoprotein (HDL), low-density lipoprotein (LDL), triglycerides (TG), total bilirubin, direct bilirubin, blood urea nitrogen (BUN), creatinine, uric acid (UA), and random blood sugar (RBS).

### 2.4. Questionnaire Survey

We questioned all participants to identify correlations between* C. sinensis* infection and known risk factors. The questionnaire was given to all participants verbally during an interview by a trained worker in a public health center. The following general information was collected: participant gender, age, current residence, and whether they consumed alcohol. We also asked questions about clonorchiasis risk-related behaviors: Did the patient have a history of eating raw freshwater fish, and if so when, where was the fish from, and what species of fish was consumed? Did the patient have a previous history of* C. sinensis* infection, or of bile duct or liver diseases, or of cooking freshwater fish? Had the patient ever experienced symptoms such as anorexia, indigestion, jaundice, abdominal discomfort, or anemia?

### 2.5. Statistical Analysis

Statistical analyses were performed using the SPSS 18.0 program (ver. 18.0; Chicago, Illinois, USA). Only participants with complete records were included in the final analysis. A chi-square test and independent *t*-test were used to check for statistically significant differences between questionnaire answers from the infected versus noninfected groups. In addition, we confirmed that the differences between the groups' laboratory results for peripheral blood cells were statistically significant, by an independent *t*-test. Differences were considered statistically significant when *p* values were less than 0.05 (*p* < 0.05). Finally, a Pearson correlational analysis was performed to compare EPG to each of the laboratory test results for infected patients.

### 2.6. Ethical Aspects

The study protocol was approved by the Ethics Committee of the Korea National Research Institute of Health, Centers for Disease Control & Prevention, Republic of Korea (2013-06EXP-04-R). All participants and local health center workers were informed of the study's purpose and procedures. Written informed consent for the research was obtained from all participants prior to enrolment. The participants found to have intestinal parasitic infections were treated with praziquantel (2-(cyclohexanecarbonyl)-3,6,7,11b-tetrahydro-1H-pyrazino[2,1-a]isoquinolin-4-one) and other appropriate antiparasitic drugs at the end of the survey.

## 3. Results

### 3.1. Infection Status

During the study period, 3,167 fecal samples were examined by the formalin-ether concentration technique (FECT). The total intestinal parasite infection rate of was 12.2% (385 cases) including 379 single infections (353 with* C. sinensis, *24 with* Metagonimus yokogawai, *four with* Trichuris trichiura*, two with* Gymnophalloides seoi*, and two with* Giardia lamblia*) and six coinfections (two or more parasite species in a single sample). Coinfections with* C. sinensis* and* M. yokogawai *represented 0.2% (six cases) of the total infections. The* C. sinensis* infection rate was 11.1%, and the mean EPG value was 171.7 ± 513.4 (Table 2). The river-adjacent village of Dongyi-myeon had the highest infection rate (20.8% with a mean EPG 262.4 ± 774.5). The second highest infection rate was 14.0% (mean EPG 58.5 ± 82.6) in the village of Annam-myeon. The lowest infection rate was 6.0% (mean EPG 95.3 ± 186.7) and was observed in the Gunseo-myeon village. The prevalence of* C. sinensis* infection was shown to be highest among patients aged 70–79 years (10.7%).

### 3.2. Basic Characteristic

The results of the fecal examinations are summarized in Table 3. The infection (egg-positive) rates were significantly higher in males (20.3%, *p* < 0.001) and alcohol drinkers (15.5%, *p* < 0.001) than in females (5.4%) and nonalcohol drinkers (7.9%), respectively. Based on the clonorchiasis risk-related behavioral questions, infection rates were significantly higher (*p* < 0.001) among the inhabitants who had eaten raw freshwater fish (14.7%) or had cooked freshwater fish (15.1%) than those who had not. In addition, significantly higher infection rates (18.3%, *p* < 0.01) were found among inhabitants whose anamneses included bile duct or liver related diseases than among those who reported no history of such diseases (10.7%). However, the infection rates did not differ significantly based on patient age, history of* C. sinensis *infection, or symptom experience.

### 3.3. Correlation between* C. sinensis* Infection and Risk Factors

To assess the relationship between* C. sinensis* infection and risk factors, we selected factors that showed significant differences between infected and uninfected groups and then analyzed the results by logistic regression. The increased odds of infection (odds ratios (ORs)) for patients with various characteristics are shown in Table 4, along with their respective 95% confidence intervals (CIs). Males, alcohol drinkers, raw freshwater fish consumers, those who had cooked freshwater fish, and those with an anamnesis including bile duct or liver disease were 4.45, 2.14, 1.64, 1.73, and 1.87 times more likely to be infected, respectively.

### 3.4. Laboratory Findings

**Figure 2:**
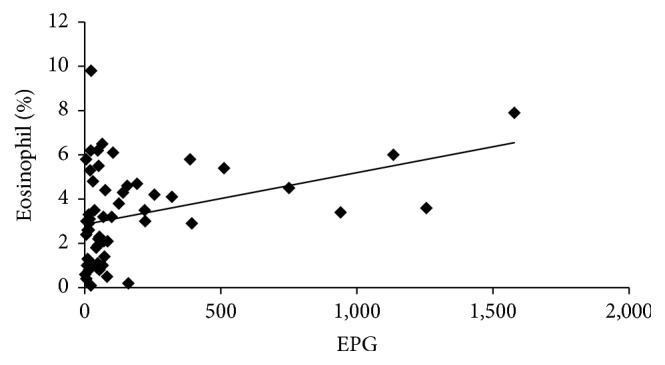
Relationship between parasite quantity (EPG) and eosinophil count in* C. sinensis* infected patients. Pearson correlation analysis shows a significant positive correlation in* C. sinensis* infected patients between the eggs per gram (EPG) in their stool and their eosinophil counts (Pearson's *r* = 0.36, *p* = 0.014, and *n* = 57). The results of linear regression analysis are shown (line slope is *y* = 0.002*x* + 2.855 and *r*^2^ = 0.13).

Among the hematological results, the MCV, MCHC, and the percentages of eosinophils (EOS) and neutrophils (NEUT) were significantly different (*p* < 0.05) between the infected and uninfected groups. However, the RBC, WBC, platelet, basophil, monocyte, and lymphocyte counts were not significantly different nor were the PDW, MCH, MPV, Hb, or Hct results. The biochemical tests revealed that LDH and ALP were mildly elevated, whereas the total protein, albumin, TG, and RBS were significantly decreased in the infected group. However, levels of AST, ALT, CPK, *γ*-GTP, total cholesterol, HDL, LDL, total bilirubin, direct bilirubin, BUN, creatinine, and uric acid were not significantly different between the groups (Table 5). The Pearson correlation analysis only revealed a statistically significant correlation (*p* = 0.014) between EPG and eosinophil count (Figure 2).

## 4. Discussion

We surveyed the characteristics and laboratory test results of patients with* C. sinensis*, living near the downstream portion of the Geum River basin. The overall prevalence of* C. sinensis* was 11.1% (353 cases). The infection rate was higher than previously reported. The prevalence of clonorchiasis in Geum River basin, for example, was reported to be 9.3% in 2002 [17] and 7.6% in 2007 [11], respectively. Among the examined villages, the infection rate was high in inhabitants within 5 km of the riverside in areas such as Dongyi-myeon (20.8%).

The clinical manifestations of the infection depend on the number of flukes, and the egg counts (EPG) give an indirect measure of the intensity of the parasitic infection. The EPG results in our study were lower than those previously reported from inhabitants of the Geum River basin in 1981 [9] and 2002 [17]. Our results indicate that over the last 30 years the infection intensity (EPG) has decreased, but infection rate has increased. These results suggest that* C. sinensis *infections are still prevalent along the Geum River basin.

The general pattern of* C. sinensis* infection frequency shows a peak in the 50–59-year-old age group [10]. However, our results showed that the infection rate was highest in those aged 70–79 years. This peak in infection frequency among people in their seventies is likely to result from an accumulation of reinfection events or chronic infection with* C. sinensis*. The likelihood of* C. sinensis *infection was much higher for males than for females. In addition, the odds of infection were higher for alcohol drinkers, natural raw freshwater fish consumers, those who cooked freshwater fish, and those with a history of bile duct or liver diseases. Some of the results of our study are similar to those of previous researchers. In previous studies, the prevalence of* C. sinensis *has been higher in males, those who live in rural areas, those who have lived for long periods of time in riverside areas, and those who have eaten raw freshwater fish [4, 17–20].

The laboratory blood tests performed here are often included as part of a regular medical checkup to look for changes in patient health. In a previous report, laboratory tests showed eosinophilia with elevated ALP in a* C. sinensis* infected group, but other parameters such as AST, ALT, *γ*-GTP, and total bilirubin were not significantly affected [16]. Liao et al. (2006) demonstrated that ALP is increased in patients with liver or bile duct disease [21]. These results were similar to our findings that the mean ALP, LDH, and eosinophil counts were significantly increased. We also confirmed a correlation between infection intensity (EPG) and eosinophil counts. Among our 33 laboratory tests, the eosinophil count was the only test whose results significantly correlated with the EPG.

## 5. Conclusions

In conclusion, our report confirms that there are risk factors of* C. sinensis* infection and that several standard laboratory test results significantly differ between infected and uninfected groups. The inhabitants residing in Geum River basin should pay attention to clonorchiasis, and further work should be focused on the early diagnosis and treatment of patients with clonorchiasis because most patients infected with* C. sinensis* do not have symptoms.

## Figures and Tables

**Table 1:** Distribution of the participants.

Location	Gender	Number of inhabitants examined	Age							
			0–19	20–29	30–39	40–49	50–59	60–69	70–79	80≤
Okcheon-eup	Male	213	4	3	8	13	28	42	90	25
	Female	389	2	1	5	8	36	88	183	67
	Total	602	6	4	13	21	64	130	273	92
Annae-myeon	Male	136	0	0	1	9	23	42	48	13
	Female	188	2	0	3	8	31	44	82	18
	Total	324	2	0	4	17	54	86	130	31
Annam-myeon	Male	76	0	0	0	2	9	12	39	14
	Female	145	0	0	1	1	14	27	78	24
	Total	221	0	0	1	3	23	39	117	38
Cheong san-myeon	Male	162	0	0	1	6	25	50	70	10
	Female	273	0	0	3	6	41	71	119	33
	Total	435	0	0	4	12	66	121	189	43
Cheong seong-myeon	Male	165	0	0	2	6	18	41	83	15
	Female	236	0	0	0	4	28	58	103	43
	Total	401	0	0	2	10	46	99	186	58
Dongi-Myeon	Male	146	0	1	6	11	22	29	61	16
	Female	190	0	1	3	13	25	46	80	22
	Total	336	0	2	9	24	47	75	141	38
Gunbuk-Myeon	Male	125	1	2	6	9	18	24	53	12
	Female	175	2	3	2	11	15	40	79	23
	Total	300	3	5	8	20	33	64	132	35
Gunseo-myeon	Male	79	0	0	1	3	11	22	33	9
	Female	137	0	0	2	3	17	26	65	24
	Total	216	0	0	3	6	28	48	98	33
Iwon-myeon	Male	125	2	1	4	4	24	28	48	14
	Female	207	1	0	4	5	28	58	91	20
	Total	332	3	1	8	9	52	86	139	34
Total	Male	1,227	7	7	29	63	178	290	525	128
	Female	1,940	7	5	23	59	235	458	880	273
	Total	3,167	14	12	52	122	413	748	1,405	401

**Table 2:** Infection rates of *C. sinensis *according to surveyed area.

Villages	Number of inhabitants examined	Infected (%)	Mean EPG ± SD
Okcheon-eup	602	48 (8.0)	94.6 ± 186.7
Annae-myeon	324	42 (13.0)	123.8 ± 267.6
Annam-myeon	221	31 (14.0)	58.5 ± 82.6
Cheongsan-myeon	435	43 (9.9)	221.3 ± 621.4
Cheongseong-myeon	401	43 (10.7)	344.0 ± 736.1
Dongi-myeon	336	70 (20.8)	262.4 ± 774.5
Gunbuk-myeon	300	33 (11.0)	31.4 ± 41.0
Gunseo-myeon	216	13 (6.0)	95.3 ± 186.7
Iwon-myeon	332	30 (9.0)	136.2 ± 200.6
Total	3,167	353 (11.1)	171.7 ± 513.4

**Table 3:** *C. sinensis* infection rates based on sociodemographic and behavioral characteristics.

Variables	Infected (%)	Uninfected (%)	Total (%)	*p* value
Number	353 (11.1)	2,814 (88.9)	3,167 (100)	
Gender				0.000
Male	249 (20.3)	978 (79.7)	1,227 (100)	
Female	104 (5.4)	1,836 (94.6)	1,940 (100)	
Age (yr)				0.146
≤49	15 (7.5)	185 (92.5)	200 (100)	
50–59	55 (13.3)	358 (86.7)	413 (100)	
60–69	89 (11.9)	659 (88.1)	748 (100)	
≥70	194 (10.7)	1,612 (89.3)	1,806 (100)	
Alcohol drinking				0.000
Yes	211 (15.5)	1,152 (84.5)	1,363 (100)	
No	142 (7.9)	1,662 (92.1)	1,804 (100)	
Raw freshwater fish consumption				0.000
Yes	147 (14.7)	851 (85.3)	998 (100)	
No	206 (9.5)	1,963 (90.5)	2,169 (100)	
History of cooking freshwater fish				0.000
Yes	149 (15.1)	837 (84.9)	986 (100)	
No	204 (9.4)	1,977 (90.6)	2,181 (100)	
Symptom experience				0.975
Yes	126 (11.2)	1,002 (88.8)	1,128 (100)	
No	227 (11.1)	1,812 (88.9)	2,039 (100)	
History of *C. sinensis *infection				0.061
Yes	38 (14.8)	218 (85.2)	256 (100)	
No	315 (10.8)	2,596 (89.2)	2,911 (100)	
History of bile ducts or liver disease				0.002
Yes	31 (18.3)	138 (81.7)	169 (100)	
No	322 (10.7)	2,676 (89.3)	2,998 (100)	

**Table 4:** Odds ratios (OR) and the 95% confidence intervals (CIs) for *C. sinensis* infection for patients with different characteristics.

Variables		OR	95% CI
Gender			
Female	Reference		
Male		4.495	3.530–5.723
Alcohol drinking			
No	Reference		
Yes		2.144	1.711–2.686
Raw freshwater fish consumption			
No	Reference		
Yes		1.646	1.313–2.064
History of cooking freshwater fish			
No	Reference		
Yes		1.725	1.376–2.163
History of bile duct or liver disease			
No	Reference		
Yes		1.867	1.243–2.803

**Table 5:** Comparison of laboratory findings between *C. sinensis* infected and uninfected group.

Laboratory findings	Infected group (*n* = 57)	Uninfected group (*n* = 40)	*p* value
	Mean ± SD	Mean ± SD	
Hematological parameters			
MCV (fL)	94.7 ± 7.1	92.0 ± 4.1	0.032
MCHC (g/dl)	33.7 ± 1.3	34.5 ± 0.8	0.001
EOS (%)	3.3 ± 2.1	2.3 ± 1.9	0.022
NEUT (%)	46.4 ± 10.9	51.2 ± 10.5	0.035
Biochemical parameters			
LDH (IU/L)	199.3 ± 51.4	176.7 ± 40.4	0.023
ALP (IU/L)	37.9 ± 12.5	30.7 ± 9.6	0.003
Total protein (g/dl)	7.2 ± 0.8	8.5 ± 2.0	0.000
Albumin (g/dl)	3.8 ± 0.2	4.0 ± 0.2	0.008
TG (mg/dl)	141.3 ± 90.3	202.6 ± 102.7	0.003
RBS (mg/dl)	111.4 ± 26.6	125.0 ± 34.3	0.040

## Abbreviations

FECT: Formalin-ether concentration technique
EPG: Eggs per gram
EDTA: 2-[2-[bis(carboxymethyl)amino]ethyl-(carboxymethyl)amino]acetic acid
SST: Serum-separating tube
Hb: Hemoglobin
Hct: Hematocrit
RBC: Red blood cell count
WBC: White blood cell count
PDW: Platelet distribution width
MCV: Mean corpuscular volume
MCH: Mean corpuscular hemoglobin
MCHC: Mean corpuscular hemoglobin concentration
EOS: Percent eosinophils
BASO: Percent basophils
MONO: Percent monocytes
NEUT: Percent neutrophils
MPV: Mean plasma volume
Olympus AU 400: Automatic blood analyzer
AST: Aspartate aminotransferase
ALT: Alanine aminotransferase
LDH: Lactate dehydrogenase
ALP: Serum alkaline phosphatase
CPK: Creatine phosphokinase
*γ*-GTP: *γ*-glutamyltranspeptidase
HDL: High-density lipoprotein
LDL: Low-density lipoprotein
TG: Triglycerides
BUN: Blood urea nitrogen
UA: Uric acid
RBS: Random blood sugar.
